# Crossing the Brooklyn Bridge: a health literacy training partnership before and during COVID-19

**DOI:** 10.5195/jmla.2021.1014

**Published:** 2021-01-01

**Authors:** Antonio P. DeRosa, Caroline Jedlicka, Keith C. Mages, Judy Carol Stribling

**Affiliations:** 1 apd2004@med.cornell.edu, Samuel J. Wood Library and C.V. Starr Biomedical Information Center, Weill Cornell Medicine, New York, NY; 2 cmj4001@med.cornell.edu, Samuel J. Wood Library and C.V. Starr Biomedical Information Center, Weill Cornell Medicine, New York, NY; 3 kcm2001@med.cornell.edu, Samuel J. Wood Library and C.V. Starr Biomedical Information Center, Weill Cornell Medicine, New York, NY; 4 jcs2002@med.cornell.edu, Samuel J. Wood Library and C.V. Starr Biomedical Information Center, Weill Cornell Medicine, New York, NY

## Abstract

**Background:**

A request for consumer health information training for public librarians led to the development of a specialized consumer health reference and health literacy training program by professional consumer health librarians from an academic medical center. Professional consumer health librarians created an interactive presentation aimed at improving public librarians' ability to respond to consumer health questions and provide vetted health resources.

**Case Presentation:**

Building on professional expertise, librarians at Weill Cornell Medicine developed a live class demonstration accompanied by a representative subject LibGuide to support public librarians who assist patrons with health questions. Skills involved in effectively communicating with patrons who are seeking consumer health information include conducting reference interviews, matching patrons' needs with appropriate resources, teaching useful Internet search methods, assessing health information, and understanding health literacy issues. Originally envisioned as two in-person live demonstrations, the team proactively adapted the program to respond to the stay-at-home social-distancing order put in place in response to the coronavirus disease 2019 (COVID-19) pandemic.

**Conclusions:**

The team successfully led an in-person live training session followed by an adapted online training experience, the latter designed to complete the curricula while complying with city and state orders.

## BACKGROUND

A public library's medical reference collection, access to online health information resources, and dedicated library professionals are vital links to community-based health information [1]. As community hubs, public libraries serve as places to enhance individual well-being and overall community health [2]. Users may consult public librarians at any point along their health care journey or information search processes. They may be looking for information for themselves, family members, or friends, and each has different levels of health literacy [3]. In the United States, over eighty million adults have basic or below literacy skills, and nine out of ten adults lack the basic skills to manage their health and prevent disease [4].

In early 2020, Weill Cornell Medicine (WCM) librarians were approached via cold-call by a Brooklyn Public Library (BPL) managing librarian to present two sessions of consumer health information training to BPL librarians and other front-facing employees. The development of content and design was left to the discretion of WCM librarians. The BPL system has a proven record of community health involvement through hosting and promoting public health fairs, raising awareness of consumer health information needs, and providing special health-related programs for the community and youth [2, 5, 6].

Acting as consultants at the request of the BPL, WCM librarians sought and received funding from the Network of the National Library of Medicine (NNLM) Middle Atlantic Region (MAR) to support a consumer health program in the BPL system. The team successfully secured a Health Information Outreach Award: “Crossing the Brooklyn Bridge: Weill Cornell Medicine Librarians Partner with the Brooklyn Public Library.” Funding supported three major goals of the project: protected time for curricular development, development of a project-specific informational banner exhibit to be given to the BPL, and creation and printing of a curated tri-fold brochure with consumer health information resources.

Initially planned as a three-hour session to be given at two different BPL branch locations on March 4, 2020, and April 8, 2020, a statewide stay-at-home social-distancing order issued by New York State Governor Andrew Cuomo on March 16, 2020, preempted the second live session. This preemption challenged WCM librarians to reenvision the workshop as a virtual event.

## CASE PRESENTATION

Understanding the potential for this collaboration to further knowledge and awareness of consumer health information, WCM librarians worked to create an interactive consumer health literacy educational workshop for public librarians. WCM consumer health librarians (DeRosa and Stribling) met early in January 2020 to define content and discuss potential dates. Two essential learning points emerged from that meeting: (1) special skills are required to conduct successful health reference interviews with health consumers, and (2) delivery of information to consumers at appropriate health literacy levels is vital for successful communication. Realizing the importance of the project and the value that additional team members would bring, two other WCM librarians (Jedlicka and Mages) were invited to join the project. Together, the team developed the following learning objectives for participants:

1. Participants will learn skills to understand the unique needs of health care consumers.
2. Participants will administer a literacy screening tool in their everyday interactions with health care consumers.
3. Participants will challenge their unconscious biases to support diverse health care consumer populations.
4. Participants will define health literacy and explain the importance of health literacy initiatives.

**Figure 1:**
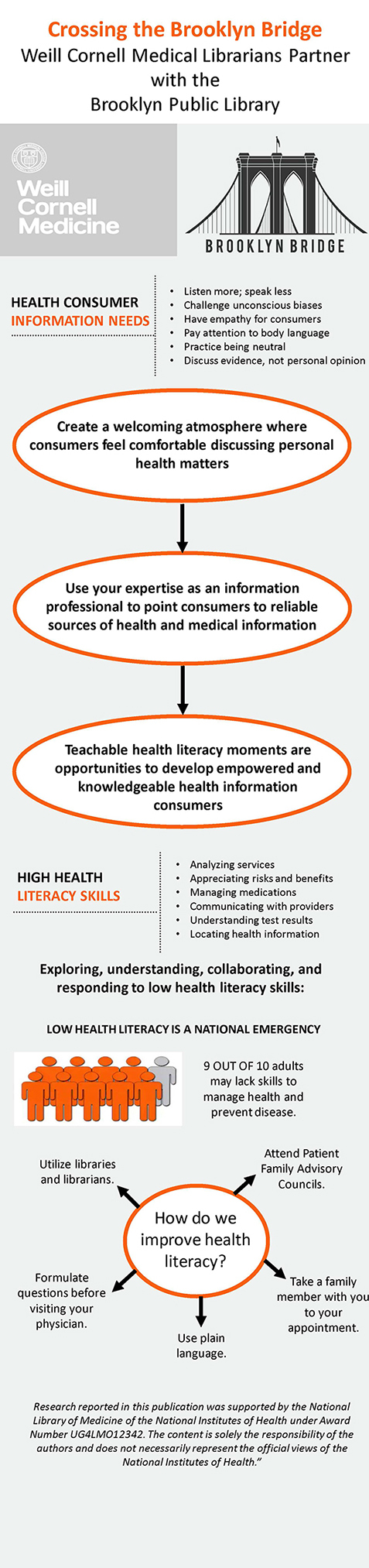
“Crossing the Brooklyn Bridge” banner

**Figure 2:**
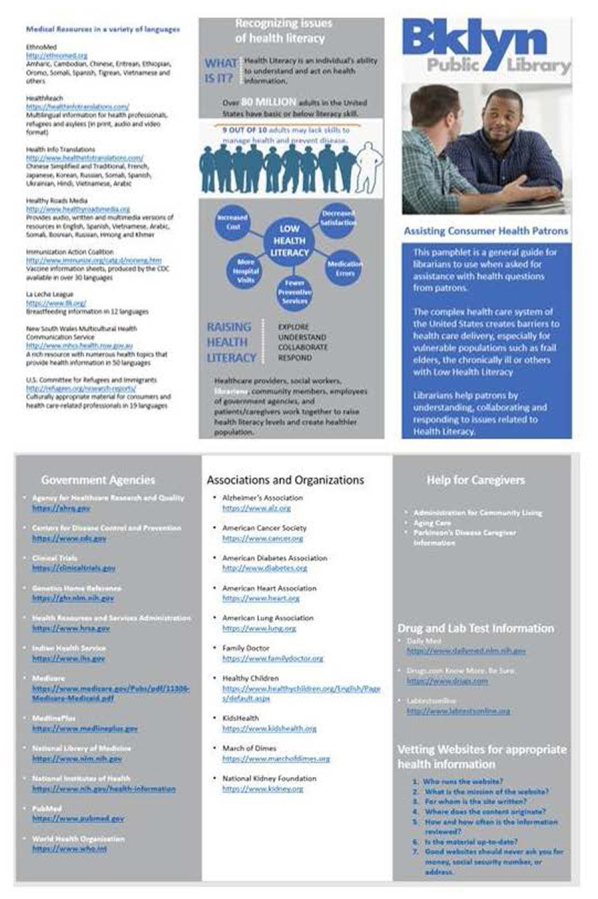
Brooklyn Public Library tri-fold brochure

Patterned in the style of the National Library of Medicine's typical traveling banner exhibits, the project's banner presented a visually impactful single-panel overview of consumer health information needs and health literacy (Figure 1). Funding also provided for printing 300 copies of a tri-fold brochure that contained a curated collection of refereed, specialized health resources from government agencies, national health organizations, and websites that specialize in multilingual consumer health information (Figure 2).

The first session held at the Bedford Branch of the BPL was presented in person, in the agreed-upon three-hour format. The session was held during morning hours, on a time and day specifically recommended by BPL collaborators. Because the library was closed to the public during the scheduled time, the BPL provided the physical space and equipment necessary for the workshop. The session took place in the library's community room, furnished with tables, laptop computers, and audiovisual equipment. The “Crossing the Brooklyn Bridge” banner was installed to complement the workshop content. Additionally, copies of the brochure with session-specific health information resources, along with MedlinePlus and All of Us brochures, were provided in bulk for participants to take back to their respective BPL branches. Content and presentation methods were discussed in detail with a BPL librarian.

### Session one, part one: “The Health Consumer Reference Interview”

The first part of the training session focused on tips and techniques for conducting reference interviews with consumers looking for health or medical information. This section featured how to assess a consumer's general literacy level, strategies for distilling salient questions from the conversation, and awareness of privacy concerns when speaking about sensitive health and medical matters.

Participants were first introduced to the most important step when conducting a reference interview on sensitive health matters: creating a welcoming atmosphere where consumers feel comfortable discussing any concerns. The importance of finding a physical, private space to speak to show respect for the health consumer and understanding and confronting unconscious biases was also addressed. Related to this, participants were reminded to maintain composure throughout the consultation and not show surprise or shock with certain questions or points of discussion. Awareness of both participants' and consumers' body language was also presented.

A fifteen-minute activity involved encouraging participants to find locations in the library conducive for conducting sensitive reference interviews with health care consumers. Participants were asked to consider and discuss the reasons they chose specific locations. Trainees were polled using the Poll Everywhere tool [7] and asked, “Where would you have a conversation about sensitive or private information?” Trainees submitted responses that were immediately populated on the presentation screen to facilitate interactive conversation. Examples of responses included: “a quiet corner of the stacks,” “the children's room during off-hours,” and “private office or conference rooms when available.” Privacy and confidentiality are hallmarks for obtaining honest answers to delicate questions. Participants were encouraged to learn to teach consumers to question their providers. This is a key step to developing vested and knowledgeable health information consumers who are empowered by information.

An instructor stressed beginning conversations with consumers by discerning the individual's general health literacy level. Participants were introduced to the Single Item Literacy Screener (SILS) as a quick and easy tool for determining the basic literacy level of an individual [8]. The SILS asks how often they need help with reading instructions, pamphlets, or other written materials provided by their doctors or pharmacies, with responses on a Likert scale ranging from 1 (never) to 5 (always). Lastly, this part of the program highlighted the importance of focusing discussions on medical evidence, not on personal opinions held by the librarian or consumer.

The reference interview was identified as an ideal time to introduce patrons to the goals of shared decision making and patient-centered care. Trainees were encouraged to teach patrons to practice shared decision making and transparency in treatment with their clinicians. Shared decision making is key for healthy outcomes. Physicians and clinicians have a duty to listen to patients, and librarians can help individuals recognize they have a right to ask questions and gear discussions with their physicians to address their personal preferences and values for treatment and care.

The capstone experience of the first portion of the workshop consisted of a mock consumer health reference interview. A worksheet and thirty-minute exercise consisted of two parts: the Consumer Health Information Request and the Mock Reference Interview (supplemental Appendix A). Each part was built upon the following clinical scenario:

“You are approached by a woman who divulges that she is forty-eight years old and has ovarian cancer. She finished her last round of chemotherapy (Carboplatin) a couple of weeks ago and is experiencing side effects of thrombocytopenia and hypomagnesemia. She is looking for research and clinical studies that discuss management of these side effects after receiving platinum-based chemotherapy (again, Carboplatin).”

### Session one, part two: “Health Literacy and Consumer Health”

Part two of the workshop featured discussions on the nature and importance of health literacy, an individual's ability to understand and act on health information, and an overview of important online consumer health resources, Participants were reminded that this may be as simple as a patron knowing *what* medications they are taking and *why*, or the complexity of a diagnosis and how it will affect the patient, their family, and their quality of life. Other points identified that individuals with low health literacy may have trouble reading and understanding food labels, completing health forms, measuring medications, speaking with health care providers about their symptoms, or following self-care instructions [4].

An overview of recent public health research illustrated the importance of health literacy and the issues that low health literacy generates for both patients and the US medical system. Discussions revolved around traditionally underserved populations who have low health literacy and those who do not usually receive preventive care. Examples included poor women who do not receive annual breast and gynecological exams, African American men who do not receive annual prostate exams, immigrants who may be frightened to interact with heath care professionals, older individuals who have multiple chronic conditions, and individuals who are uninsured [9–12]. Participants were encouraged to find creative ways to leverage local connections with neighborhood community centers, churches, or other community groups to raise health literacy levels in their communities.

The transformative shift of health information from primarily hard copy to electronic formats creates another type of literacy barrier for users: technological literacy. Using electronic resources requires familiarity with computer technology, often another challenge for patrons with low health literacy [13]. Public librarians not only need to devote ample time to assist individuals with low levels of technological literacy, but also must connect these patrons to vetted and reliable health resources located online.

### Workshop interrupted: COVID-19 and a virtual space for session two

The jarring emergence of COVID-19 and the New York State–mandated order for social distancing precluded the second in-person training session and required WCM librarians to adapt their program. Like thousands of workers all over the world, WCM librarians worked remotely while reformatting the workshop. NNLM MAR and the BPL graciously allowed flexibility for WCM librarians to revise the second session. Zoom-based workshops held in real-time were rejected for a variety of reasons including potential instability of the personal home technology of participants and the need to meet the project deadline. The WCM team researched asynchronous options and ultimately redesigned the workshop as a publicly available LibGuide that provided all content from the live session as well as additional information about the emerging corona virus [14]. This enabled BPL librarians to participate remotely in the virtual platform at their leisure. The format added benefits of making the training available to a larger audience as the LibGuide was not restricted to specific instructions or individuals.

Using Zoom, WCM librarians recorded original lectures from session one and embedded those videos in the workshop LibGuide. The first video, “A Health Consumer Walks into a Library: Techniques for Handling the Reference Interview for Health and Medical Information,” was posted along with a portable document format (PDF) of the consumer health reference interview worksheet for participants to complete. Although this did not recreate the immediacy of a group experience, interested participants completing the exercise were invited to email the worksheets to a WCM librarian (DeRosa) to provide feedback. The second video, “Techniques for Improving Health Literacy,” was posted along with links to online tools for creating consumer-friendly health resources, such as the Gunning Fog Index and the Patient Education Materials Assessment Tool [15, 16].

A notable update in the virtual training was the inclusion of significant numbers of COVID-19 resources in the LibGuide. As COVID-19 emerged as a full-blown pandemic, with New York City hit especially hard, there was a critical need for vetted, reliable public health information on the novel coronavirus. To assist participants in answering consumer health questions, WCM librarians identified more than twenty COVID-19 consumer-friendly resources. Additionally, the LibGuide included a page of general consumer health resources and information for consumers about shared decision making and transparency in treatment.

The last section of the workshop LibGuide features a course-completion survey for program evaluation (supplemental Appendix B). Created with Qualtrics software, the survey collects participants' perceptions of the quality, quantity, and usefulness of the various components of the online workshop and will inform future iterations of this experience. WCM librarians plan to make the LibGuide a permanent offering, although in the future, the name will be generalized to enhance the workshop's applicability to all interested public health librarians.

## DISCUSSION

The emergence of the COVID-19 pandemic radically shifted the routines of WCM librarians and drastically changed the program mid-stream. The team adopted an asynchronous training module to address the inability to deliver live demonstrations and instructions. COVID-19 also dramatically changed the culture of health care and heightened the need for vetted, reliable health information.

Health and hygiene are daily topics of discussion and the public is inundated with often contradicting messages related to COVID-19 prevention and treatment. Because public librarians remain on the frontline of consumer health questions, whether they work with patrons in-person or via virtual methods, the importance of appropriately identifying patron needs and providing reputable health information is increasingly important. Successful patron transactions lead to the development of trust and rapport, both essential to establishing partnerships between community members and public librarians.

Medical library professionals and public librarians must continue to work together to empower patrons to locate reliable health information and to raise health literacy levels in the United States. Successful collaborations may take the form of the workshop introduced in this case study or may follow earlier examples described by Danhoundo [17] or Koos [18]. Overall, we found our partnership with the BPL with the assistance of NNLM MAR to be impactful and professionally satisfying. The program underscored the importance of seeking partnerships and the potential of collaborations enabling dynamic outreach and novel funding opportunities, all while managing the complexities of remote work and adaptive learning techniques during a global pandemic. Preserving bonds between professional health sciences librarians and public library colleagues is increasingly important. Further cultivation of this connection will use the information gained from this workshop, via informal feedback and the online survey results, to inform and refine future health literacy-focused workshops.

This program was not without its challenges. Inherent difficulties in scheduling programming between two large organizations was apparent. There was much back and forth to find mutually convenient dates between both institutions. After scheduling the first session, BPL scheduled an impromptu system-wide rally that further complicated scheduling. Additionally, we have not received as many responses to our virtual training session course-completion survey as we had originally anticipated. This may be explained due to the inherent difficulty in promoting a survey via a prerecorded, asynchronous training session embedded in a static LibGuide page. Furthermore, page views have plateaued since we published the LibGuide in April. As BPL branches are now reopening with limited services, it is more important than ever that BPL librarians are up to speed on reputable consumer health resources and practices. We will reach out to our liaisons at the BPL to remind them of our availability to provide assistance and of the LibGuide's content and pertinence as the pandemic continues to evolve.

As of this writing, successful vaccination for the highly contagious COVID-19 disease is not within immediate reach; therefore, all librarians and educators in academic and public roles must continue adjusting new training methods for health consumers. This challenge represents a real opportunity for increased sharing of information between academic and public librarians. Refining, developing, and understanding the technical elements of online teaching methods are essential for success in the “new normal” world in which we live. Disadvantages of this type of instruction include an inability to form strong personal connections with learners or to “read” the room for possible misunderstanding or confusion about the material. Advantages of the model include a permanent electronic space for the information that users can visit as often as necessary to master the concepts. The electronic format assures public library managers that all their staff have access to the same information: nothing is omitted or added from class to class. The added value of this model is more information can be shared by medical academic librarians with their public librarian counterparts around the country.

## Data Availability

There are no data associated with this article.

## SUPPORTING AGENCY

Network of the National Library of Medicine Middle Atlantic Region.

## SUPPLEMENTAL FILES

**Appendix A:** Reference interview worksheet

**Appendix B:** Survey
