# Supplementary material for: Crossing the Brooklyn Bridge: a health literacy training partnership before and during COVID-19
Source: J Med Libr Assoc. 2021 Jan 1;109(1):90–6. doi: 10.5195/jmla.2021.1014 (PMC7772982; doi:10.5195/jmla.2021.1014)
Supplement: Supplementary file 1 — Appendix A: Reference interview worksheet [file jmla-109-1-90-s01.pdf]

JMLA Journal of the Medical Library Association

SC

Supplemental content to
J Med Libr Assoc. Jan;109(1):dx.doi.org/10.5195/jmla.2021.1014
www.jmla.mlanet.org
© DeRosa, Jedlicka, Mages, Stribling 2021

Crossing the Brooklyn Bridge: a health literacy training partnership before and during COVID-19

Antonio P. DeRosa, AHIP; Caroline Jedlicka, AHIP; Keith C. Mages, AHIP; Judy Carol Stribling, AHIP

APPENDIX A

Reference interview worksheet

Part I: Consumer health information request

The scenario:

You are approached by a woman who divulges that she is forty-eight years old and has ovarian cancer. She finished her last round of chemotherapy (Carboplatin) a couple of weeks ago and is experiencing side effects of thrombocytopenia and hypomagnesemia. She is looking for research and clinical studies that discuss management of these side effects after receiving platinum-based chemotherapy (again, Carboplatin).

1. Rank the following in sequential order (1=the first thing you would do; 7=the last thing you would do):

___ Provide printed material and/or email consumer links to resources

___ Ask questions for clarity and determine salient points

___ Demonstrate reliable and trusted online resources for finding health and medical information

___ Set expectations: you are not a health care professional, but more of a health advocate or educator to assist consumers in staying informed to improve their knowledge and understanding of health and medical conditions

___ Find a private place to speak

___ Listen to consumer's concerns and needs

___ Assess the general literacy level of the consumer

2. The consumer has thanked you for your help and the information you have provided. She then asks whether or not she should continue with chemotherapy treatment. What do you do?

____

3. Would you utilize the Single Item Literacy Screener (SILS) tool for assessing this patient's literacy level?

___ Yes

___ No

___ Undecided

If undecided, explain ____

JMLA Journal of the Medical Library Association

SC
Supplemental content to
J Med Libr Assoc. Jan;109(1):dx.doi.org/10.5195/jmla.2021.1014
www.jmla.mlanet.org
© DeRosa, Jedlicka, Mages, Stribling 2021

The SILS tool asks the question:
How often do you need to have someone help you when you read instructions, pamphlets, or other written material from your doctor or pharmacy?
1: Never, 2: Rarely, 3: Sometimes, 4: Often, and 5: Always
Morris NS, MacLean CD, Chew LD, Littenberg B. The single item literacy screener: evaluation of a brief instrument to identify limited reading ability. BMC Fam Pract. 2006 Mar 24;7:21.

Part II: Mock reference interview

Instructions: Using the same consumer scenario in part one, read through the mock reference interview with a partner. One of you should play the role of consumer and other should play the librarian. Discuss the issues with this mock interview. What could have been done differently? What could have been done better? What did not occur at all? List your observations in the space provided.

Consumer: Hello, I would like to find some information on a medical condition I have. Is there someone I can speak to?

Librarian: Hi there. Yes, my name is [insert name]. I would be happy to help you.

Consumer: I have cancer and am receiving a chemotherapy called Carboplatin. It has given me some specific side effects that I want to learn more about. Do you have information on thrombocytopenia and hypomagnesemia?

Librarian: Yes, give me a moment while I pull up some information for you.

Consumer: Okay.

Five minutes pass while librarian searches for information.

Librarian: Okay, I found some materials from MedlinePlus and the Mayo Clinic. Would you like me to print these out for you?

Consumer: Yes, please. Can you also email them to me? Here is my email address.

Librarian: Sure thing. I just emailed the pages to you. Let me grab the print outs from the printer.

Consumer: Thank you. Can I also find some more information on my own?

Librarian: Yes. Let me get you set up at a computer so you can do some searching.

Librarian gives consumer a ticket and shows her to a computer.

Consumer: Thank you. I will try and find some more information on my own.

Librarian: Great. Thank you for coming in today.
