# Supplementary material for: Crossing the Brooklyn Bridge: a health literacy training partnership before and during COVID-19
Source: J Med Libr Assoc. 2021 Jan 1;109(1):90–6. doi: 10.5195/jmla.2021.1014 (PMC7772982; doi:10.5195/jmla.2021.1014)
Supplement: Supplementary file 2 — Appendix B: Survey [file jmla-109-1-90-s02.pdf]

Journal of the Medical Library Association

Supplemental content to
J Med Libr Assoc. Jan;109(1):dx.doi.org/10.5195/jmla.2021.1014
www.jmla.mlanet.org
© DeRosa, Jedlicka, Mages, Stribling 2021

Crossing the Brooklyn Bridge: a health literacy training partnership before and during COVID-19

Antonio P. DeRosa, AHIP; Caroline Jedlicka, AHIP; Keith C. Mages, AHIP; Judy Carol Stribling, AHIP

APPENDIX B
Survey

Participants will be directed to rate all responses in the following manner:

1 for Strongly disagree, 2 for Disagree, 3 for Neutral, 4 for Agree, 5 for Strongly agree

1a. When thinking specifically about video #1, "A Health Consumer Walks into a Library: Techniques for Handling the Reference Interview for Health and Medical Information," the **quantity** of information presented met my expectations.

1b. When thinking specifically about video #1, "A Health Consumer Walks into a Library…," the **quality** of information presented met my expectations.

1c. When thinking specifically about video #1, "A Health Consumer Walks into a Library…," the information presented was **useful.**

1d. When thinking specifically about the reference interview worksheet, which accompanies video #1, this worksheet was **useful.**

2a. When thinking specifically about video #2, "Techniques for Improving Health Literacy," the **quantity** of information presented met my expectations.

2b. When thinking specifically about video #2, "Techniques for Improving Health Literacy," the **quality** of information presented met my expectations.

2c. When thinking specifically about video #2, "Techniques for Improving Health Literacy," the information presented was **useful.**

3a. When thinking specifically about the various health literacy and consumer health resources suggested, the **quantity** of information presented met my expectations.

3b. When thinking specifically about the various health literacy and consumer health resources suggested, the **quality** of information presented met my expectations.

3c. When thinking specifically about the various health literacy and consumer health resources suggested, the information presented was **useful.**

JMLA Journal of the Medical Library Association

SC

Supplemental content to
J Med Libr Assoc. Jan;109(1):dx.doi.org/10.5195/jmla.2021.1014
www.jmla.mlanet.org
© DeRosa, Jedlicka, Mages, Stribling 2021

3a. When thinking specifically about the COVID-19 resources suggested, the **quantity** of information presented met my expectations.

3b. When thinking specifically about the COVID-19 resources suggested, the **quality** of information presented met my expectations.

3c. When thinking specifically about the COVID-19 resources suggested, the information presented was **useful.**

4a. When thinking about this "Assisting Consumer Health Patrons" course as a whole, the **quantity** of information presented met my expectations.

4b. When thinking about this "Assisting Consumer Health Patrons" course as a whole, the **quality** of information presented met my expectations.

4c. After completing this "Assisting Consumer Health Patrons" course, I feel more capable of helping my patrons meet their consumer health information needs.

4d. Overall, the digital layout of this "Assisting Consumer Health Patrons" course met my expectations.
